# Supplementary material for: High niche diversity in Mesozoic pollinating lacewings
Source: Nat Commun. 2018 Sep 17;9:3793. doi: 10.1038/s41467-018-06120-5 (PMC6141599; doi:10.1038/s41467-018-06120-5)
Supplement: Supplementary file 1 — Supplementary Information [file 41467_2018_6120_MOESM1_ESM.pdf]

SUPPLEMENTARY INFORMATION

High niche diversity in Mesozoic pollinating lacewings

Liu et al.

Contents

Supplementary Note 1: Expanded description and taxonomic notes of Burmese amber long-proboscid psychopsoids
Supplementary Note 2: List of taxa used in the phylogenetic analysis
Supplementary Note 3: List of characters for the phylogenetic analysis
Supplementary Figures 1-14
Supplementary Tables 1-2
Supplementary References

Supplementary Note 1: Expanded description and taxonomic notes of Burmese amber long-proboscid psychopsoids

Family Kalligrammatidae Handlirsch

Subfamily Cretanallachiinae Makarkin emend. nov.

Remarks on familial position. Cretanallachiinae was established and placed in Dilaridae by Makarkin[1], including *Cretanallachius* (as the type genus of the subfamily) and *Burmopsychops*. Makarkin[1] presented a detailed morphological comparison with extant Dilaridae and other lacewing families to support the dilarid affinity of Cretanallachiinae as well as Cretadilarinae that was established as another dilarid subfamily in the same paper, and proposed two autapomorphies, i.e., the loss of ligula and the pectinate male antennae, as the arguments, although without any supports from rigorous phylogenetic analysis. However, Makarkin seemed to selectively ignore Kalligrammatidae that possess almost identical siphonate mouthparts and similar wing shape and venations at least to Cretanallachiinae[1]. Our present finding of the new lacewing genus (i.e. *Burmogramma* gen. nov.) with bipectinate male antennae, posteroventrally protruding female tergum 9, and typical kalligrammatid wing venations as well as eye spots clearly suggests the kalligrammatid affinity of these Burmese amber long-proboscid lacewing species that were previously either placed in Psychopsoidea with uncertain familial status[2] or incorrectly placed in Dilaridae[1,3].

The sexually dimorphic antennae that are pectinate in males are a well-known example of convergent evolution in various heterogeneous insect groups in relevance to the pre-mating chemical communication[4]. Thus, although the unipectinate male antenna supports the monophyly of Dilaridae[2], the pectinate male antenna, which includes the unipectinate type in Dilaridae and the bipectinate type in Cretanallachiinae, does not imply to be a synapomorphy supporting the dilarid affinity of Cretanallachiinae, but actually being evolved independently between Dilaridae and Cretanallachiinae.

The loss of ligula is also a problematic character for assigning Cretanallachiinae into Dilaridae. Makarkin[1] considered that the ligula in Cretanallachiinae is lost and interpreted the “ligula” in Lu et al.[2] to be the lacinia based on its relatively basal position in comparison to the prementum. However, in all materials we examined the prementum is actually absent and the “prementum” illustrated in Makarkin[1] is

probably artificial. Moreover, the ligula in *Fiaponeura* is unpaired with bifid apex (see Lu et al.[2]: fig. 4), which conforms to the morphological ground plan of lacewing ligula.

The presence of paired tibial spurs in all species of Cretanallachiinae is herein confirmed. Previous reports on the absence of tibial spurs in *Cretanallachius* (see Lu et al.[2]) and *Burmopsychops* (see Makarkin[1]) are possibly due to preservation matters.

Association between conspecific males and females was primarily based on the similarity of body size, wing venation, and wing marking pattern. The combination of characters of body-size, wing venations and wing marking patterns is in general stable among conspecific males and females but differs among different species. For example, *B. liui* and *F. penghiani* have very characterized wing markings and venations, which can facilitate the association of males and females. Similarly, *C. magnificus* is the smallest species of Cretanallachiinae known so far and has no wing marking. Therefore, the newly found female of this species was linked to the male by its very small body-size (smaller than females of any other cretanallachiine species) and the immaculate wings.

The male and female genitalia of Cretanallachiinae also show sufficient morphological differences as a distinct family-level group from that of Dilaridae. Considering the male genitalia of Cretanallachiinae, Makarkin[1] disagreed with the interpretation of the paired large valvate lobes to be the gonocoxites 9[2], but alternatively considered it to be a part of enlarged tergum 9 as an argument supporting close relationship between this subfamily and Dilarinae. Although in most extant families of Neuroptera (except Nevrorthidae, Sisyridae and some species of Osmylidae) the male gonocoxites are present as small internal sclerites, the condition of this genital sclerite in fossil lacewings, particularly in extinct families/subfamilies is poorly known, and the possibility of the presence of large external male gonocoxites 9 in some extinct lacewing families cannot be excluded. Based on our examination of males of several cretanallachiine species, there is no doubt that these large paired lobes are gonocoxites 9, which are present at the typical position of male gonocoxites 9 (i.e., a position posteroventrad tergum 9) of Neuroptera, while the true male tergum 9 in Cretanallachiinae is a short and arched sclerite posterodorsally connected with paired ectoprocts, which is also a typical feature in Neuroptera. The female genitalia of Cretanallachiinae are highly specialized considering the tergum 9 with a pair of valvate posteroventral lobes that largely envelope the gonocoxites 9.

Furthermore, the female ectoprocts are paired and largely associated with the dorsal part of tergum 9, and there is a small hole (putatively homologized with the copulatory fovea in Psychopsidae; see Oswald[5] and Bakkes et al.[6]) anteriad gonocoxites 8 (discernible in a female of *Oligopsychopsis groehni* (Makarkin)). Notably, these remarkable characters, although with some modifications, are present in extant Psychopsidae (see Oswald[5]: figs. 43, 49). However, Makarkin[1] just gave a groundless conclusion that the ovipositor (= gonocoxites+gonostyli 9) of Psychopsidae and Cretanallachiinae were independently evolved and considered an Osmylid-like feature of the ovipositor in Cretanallachiinae. Based on our examination, the female gonostyli 9 are just present on subdistal portion of ventral surface of gonocoxites 9, which was mentioned as the feature of Psychopsidae by Makarkin[1]. Nevertheless, the most important arguments supporting the closer relationship between Cretanallachiinae and Psychopsidae rather than Dilaridae refer to the aforementioned similar characters of female tergum 9, ectoprocts and gonocoxites 8.

Finally, the result of our phylogenetic analysis suggests the kalligrammatid affinity of Cretanallachiinae based on the reduction of mandibles (char. 24:1), the subtriangular branching field of forewing MP (char. 11:1), and the presence of eyespots (char. 17:1), although in some small-sized cretanallachiine species the forewing MP branches are reduced, and the eyespots are feebly developed or even lost.

Genus *Burmopsychops* Lu, Zhang & Liu, 2016

Remarks. This genus appears to be closely related to *Cretanallachius* in having similar immaculate wings, barely branched MA vein, and absence of nygmata, but it can be separated from the latter based on the forewing MA separating from R and the absence of sigmoid stem of hind wing MA.

***Burmopsychops limoae* Lu, Zhang & Liu, 2016**

(Fig. 1 and Supplementary Fig. 2)

Revised diagnosis. Almost same as for the genus. Forewing length 7.7–10.0 mm.

Re-description. See Lu et al.[2] for the description of female non-genital characters. Male unknown. Female genital segments: Gonocoxites 8 seemly paired; a small sclerite (putative gonapophysis 8) present posteriad gonocoxites 8. Tergum 9 distinctly separated into a pair of broad lateral valves, with dorsal part reduced and

membranous; lateral valves strongly protruding posteriad and slightly curved dorsad, with rounded apices. Gonocoxites 9 slightly shorter than and largely enveloped by posterolateral lobes of tergum 9, distally tapering; gonostyli 9 present at subdistal portion of ventral surface of gonocoxites 9, short, digitiform. Ectoprocts paired, subtriangular; ovoid callus cerci present on posterior margin of ectoprocts.

Materials examined. Holotype: EMTG BA-001293, a complete adult female. Other material: NIGP164485, a complete adult female.

Genus *Cretanallachius* Huang et al., 2015

***Cretanallachius magnificus* Huang et al., 2015**

(Supplementary Figs. 4 and 5)

Revised diagnosis. Same as for the genus.

Re-description. See Huang et al.[3] and Lu et al.[2] for the description of male. Non-genital characters of female almost identical to male, except for the moniliform antennae.

Male genital segments: Tergum 9 short, arched, distinctly extending ventrad, dorsally membranous. Sternum 9 much longer than tergum 9, subtriangular, posteromedially with a horn-like projection. Gonocoxites 9 paired, broadly valvate, distinctly narrowed posteriad, with a few small dents on inner surface at tip. Ectoprocts paired, flatly subrectangular; ovoid calluls cerci present on posterolateral margin of ectoprocts. A pair of slenderly elongate, spinous sclerites (putative gonocoxites 10) present internally, largely enveloped by gonocoxites 9.

Female genital segments: Tergum 9 distinctly separated into a pair of broad posterolateral lobes, with dorsal part reduced and membranous; posterolateral lobes strongly protruding posteriad and slightly curved dorsad. Gonocoxites 9 slightly shorter than and largely enveloped by lateral valves of tergum 9, distally tapering; gonostyli 9 invisible due to preservation condition. Ectoprocts paired, subtriangular, with rounded apex; callus cerci absent.

Materials examined. EMTG BU-002169, an male adult (wings partly not preserved); EMTG BU-002266, a partly preserved male adult; NIGP164487, a complete adult female; CAU-BA-XF-18003, a complete adult female.

Genus *Oligopsychopsis* Chang et al., 2018

Remarks. This genus was originally erected based on a single male specimen from

the Burmese amber7. The cretanallachiine affinity of this genus is undoubted in light of the presence of bipectinate male antennae and the siphonate mouthparts. However, after our re-examination of the holotype of the genus-type species, we found that the genus was poorly described with a number of incorrect character descriptions and inaccurate drawings concerning wings in Chang et al.[7]. First, the basal forewing nygma is herein confirmed to be absent, while a median forewing nygma appears to be present although being not distinct in the holotype of *O. penniformis*. Second, the forewing MA, MP, CuA and CuP veins labeled in Chang et al.[7] (fig. 3A) are indeed the last branch of RP, the MA, the MP, and the CuA+CuP respectively. Thus, a revised diagnosis is presented above for correctly defining this genus. *Oligopsychopsis* appears to be closely related to *Cretogramma* gen. nov. in having some similar forewing characters, i.e., the presence of ovoid dark marking at middle, the presence of a median nygma, the presence of interlink veinlets among some costal crossveins, and the MA proximally separating from R. However, it can be distinguished from the latter genus by the deeply branched forewing MA and the presence of sigmoid hind wing MA stem.

***Oligopsychopsis groehni* (Makarkin, 2017) comb. nov.**

(Fig. 2g-i and Supplementary Fig. 7)

Revised diagnosis (from Makarkin[1]). Forewing length 13–14 mm in both male and female. Forewing medially with an ovoid dark marking, which bears obscure margin, and with some smaller dark spots along subcostal space; dark markings on distal half of wing very indistinct if present. Posterolateral lobe of female tergum 9 nearly 3.0 times as long as wide.

Re-description. Male (NIGP164476). Head orthognathous, short. Compound eyes large and semi-globular. Antennae bipectinate. Mouthparts siphonate. Pronotum short, slightly wider than long; meso- and metathorax robust, mesothorax ~2.0× length of metathorax. Legs slender, some spinous setae present on tibiae.

Forewing: Broadly subtriangular, with round distal margin; 13.4 mm long. An ovoid dark marking present medially, with obscure margin; some smaller dark spots present along subcostal space; dark markings on distal half of wing very indistinct if present. Trichosors present along distal margin. A nygma present at middle of wing. Costal space broad, ~4.0× width of subcostal space, with most crossveins on distal half deeply forked and with a few interlink veinlets among them; a simple humeral

veinlet slightly bent to wing base. Subcostal space slightly broader than RA space proximally but narrowed distad. Subcostal space with 19 widely spaced crossveins. RA space with 9 widely spaced crossveins. ScP and RA not fused distad. RP with 6 primary branches; posterior 3 branches deeply forked. MA separating from R, deeply and dichotomously branched. MP pectinately branched from proximal position, with 4 main branches that form a narrow, subtriangular field. CuA and CuP diverging proximad initial branching point of MP, nearly parallel with each other on proximal half, CuA with 2 short branches distally, CuP deeply and pectinately branched, with much denser branches than CuA. Anal space short and narrow; A1 deeply and pectinately branched into 4 branches; A2 bifurcate. Crossveins in general numerous, and widely spaced with each other; an outer gradate series of crossveins present.

Hind wing: Not preserved. Male genital segments: Not preserved.

Females (NIGP164477, NIGP164478, NIGP164479, and CAU-BA-XF-18002). Forewing 13.9 mm long, hind wing ~13.0 mm long. Wing characters generally same as that of male. See detailed description also in Makarkin (2017).

Female genital segments: Tergum 9 bilobed dorsoventrally; dorsal part strongly narrowed; ventral part strongly protruding posteriad, forming a pair of valvate lobes that are slightly curved dorsad, nearly 3.0 time as long as wide. A narrowly subtriangular sclerite (putative gonapophysis 8) that is acutely tapering posteriad present between bases of ventral parts of tergum 9, proximally with a small hole. Gonocoxites 9 enveloped by posterolateral lobes of tergum 9, distally narrowed; gonostyli 9 present at subdistal portion of ventral surface of gonocoxites 9, digitiform. Ectoprocts paired, proximally largely fused with dorsal part of tergum 9; callus cerci absent.

Materials examined. NIGP164476, a complete male adult. NIGP164477, a complete female adult. NIGP164478, a female adult. NIGP164479, a female adult. CAU-BA-XF-18002, a female adult. CAU-BA-WN-18001, a female adult.

Remarks. This species was originally placed in *Burmopsychops* in Makarkin[1]. However, it should be transferred to *Oligopsychopsis* based on the presence of most generic diagnostic characters of *Oligopsychopsis* in this species, such as the presence of forewing median nygma, the deeply branched forewing MA, and the presence of sigmoid stem of hind wing MA. It is unclear whether a sigmoid stem of hind wing MA is present in the holotype of *O. groehni*. However, we found this veinlet in some other specimens associated with *O. groehni*.

***Oligopsychopsis penniformis* Chang et al., 2018**

Revised diagnosis (from Chang et al.[7]). Forewing length ~21 mm in male. Fore- and hind wing almost immaculate, with only indistinct trace of median markings.

Material examined. Holotype: CNU-NEU-MA2017001, a complete male adult.

Remarks. As mentioned above in the Remarks of the genus, this species was inadequately described in Chang et al.[7] based on our re-examination of the type specimen. However, we have no chance to carefully re-describe and re-draw the holotype. The re-description of this species may be done pending anyone of the authors of this species in the future.

Family Incertae sedis

Genus *Fiaponeura* Lu, Zhang & Liu, 2016

Remarks. In previous discussion on the systematic placement of *Fiaponeura* (Lu et al.[2]), it was mentioned that this genus lacks the autapomorphy of Kalligrammatidae (i.e., the posterior branch of forewing MP with many distal pectinate branches, consisting of an expansive, triangular region). However, many species of the presently reported Burmese amber kalligrammatids also possess relatively narrow forewing MP field, suggesting that lack of the broad forewing MP field is not the evidence to disclaim the kalligrammatid affinity of *Fiaponeura*. Furthermore, the presence of siphonate mouthparts and multiple forewing ORBs, shared by *Fiaponeura* and some kalligrammatids (e.g. the species of Kallihemerobiinae Ren & Engel), imply the kalligrammatid affinity of *Fiaponeura*. The newly found male of *F. penghiani* has similar external male gonocoxites 9 that are also present in Cretanallachiinae. However, the male antennae of *Fiaponeura* are filiform. The present phylogenetic analysis thus assigned *Fiaponeura* to be the basalmost lineage of the monophyletic group comprising Kalligrammatidae and Aethogrammatidae.

***Fiaponeura penghiani* Lu, Zhang & Liu, 2016**

(Supplementary Fig. 9)

Revised diagnosis. Same as for the genus.

Re-description. See Lu et al.[2] for description of female. Non-genital characters in male almost identical to female.

Male genital segments: Tergum 9 rather short and strongly extending ventrad.

Sternum 9 relatively broad, nearly rectangular, distinctly protruding beyond posterior margin of tergum 9 in lateral view. Gonocoxites 9 present as a pair of external sclerites, distinctly prolonged and narrowed posteriad, with sharply pointed and incurved apices. Ectoprocts present, slightly longer than tergum 9. Putative gonocoxites 10 present as a large, dorsally curved sclerite.

Materials examined. Holotype: EMTG BU-001679, an almost complete adult female (wings partly not preserved). Other material: NIGP168262, an almost complete adult male (wings partly not preserved).

Supplementary Note 2: List of taxa used in the phylogenetic analysis

Family Dilaridae Newman, 1853

Genus *Dilar* Rambur, 1838

Genus *Nallachius* Navás, 1909

Family Ithonidae Newman, 1853

Genus *Rapisma* McLachlan, 1866

Family Prohemerobiidae Handlirsch, 1907

Genus *Prohemerobius* Handlirsch, 1907

Family Psychopsidae Handlirsch, 1906

Genus *Balmes* Navás, 1910

Genus *Electropsychops* Lu, Zhang, Ohl & Liu, 2017

Genus *Litopsychopsis* Engel & Grimaldi, 2008

Family Osmylopsychopidae Martynova, 1949

Genus *Daopsychops* Peng et al. 2015

Genus *Nematopsychops* Peng et al. 2015

Genus *Osmylopsychops* Tillyard, 1923

Family Kalligrammatidae Handlirsch, 1906

Genus *Kalligramma* Walther, 1904

Genus *Kallihemerobius* Ren & Oswald, 2002

Genus *Meioneurites* Handlirsch, 1906

Genus *Oregramma* Ren, 2003

Genus *Sophogramma* Ren & Guo, 1996

Family Aetheogrammatidae Ren & Engel, 2008

Genus *Aetheogramma* Ren & Engel, 2008

Family insertae sedis

Genus *Burmogramma* gen. nov.

Genus *Burmopsychops* Lu, Zhang & Liu, 2016

Genus *Cretanallachius* Huang et al. 2015

Genus *Fiaponeura* Lu, Zhang & Liu, 2016

Genus *Cretogramma* gen. nov.

Genus *Oligopsychopsis* Chang, Fang, Shih, Ren & Wang, 2018

Supplementary Note 3: List of characters for the phylogenetic analysis

Forewing:

1. Branched recurrent humeral veinlet: (0) absent; (1) present.

2. Ratio of maximum width of costal space/subcostal space: (0) no more than 3:1; (1) 4:1-5:1; (2) more than 5:1. Comments: The forewing costal space in lacewings is in general much wider than the subcostal space of the same wing, but in most cases the maximum width of costal space does not exceed 3.0× width of subcostal space. However, in some broad-winged species, such as the species of Psychopsoidea, the forewing costal space is wider than 4.0× width of subcostal space. Particularly, in Psychopsidae, the forewing costal space is over 5.0 times as wide as subcostal space.

3. Costal space: (0) narrowed distad; (1) not narrowed distad.

4. Costal crossveins: (0) mostly simple; (1) with many forked ones.

5. Interlinked veinlets between costal crossveins: (0) absent; (1) present.

6. ScP and RA: (0) not fused distad; (1) fused distad, forming a sharp angle of subcostal space; (2) fused distad by an abrupt subcostal curve, which is continuous posteriorly with a short crossvein connecting to RP. Comments: State 2 is present only in Psychopsidae.

7. Position of ScP: (0) entering wing margin proximad or within pterostigmal area; (1) entering wing margin posteriad pterostigmal area. Comments: State 1 is present in many crown groups of Neuroptera, such as Chrysopidae, Myrmeleontoidea and Psychopsoidea.

8. Number of scp-ra crossveins: (0) 1; (1) 0; (2) less than 20; (3) more than 20. Comments: The presence of only one forewing scp-ra crossvein, usually near wing base, is developed in Megaloptera, Raphidioptera and many groups of Neuroptera, and it is considered to be plesiomorphic. However, in a few species, such as the dilarid species of *Nallachius*, there is no forewing scp-ra crossvein. In Ithonidae and Psychopsoidea, the scp-ra crossveins are well developed. Particularly, in some ithonid species of *Rapisma* and some large-sized kalligrammatid species with relatively denser crossvenations, there are more than 20 forewing scp-ra crossveins, which is considered to be the most derived condition.

9. Oblique radial branches (ORB): (0) absent; (1), present, but only one; (2) present, two or more. Comments: This character refers to the configuration of forewing RP+MA. Generally, there is only one branch, i.e. the stem of forewing RP+MA, separated from R in Neuroptera. However, in some lacewing species, the stem of

forewing RP+MA is fused with R for a short or long distance. The most typical example possessing this character state is Hemerobiidae (brown lacewings). In addition, the dilarid species of *Dilar* and some species of Cretanallachiinae (e.g. *Burmopsychops*, *Oligopsychopsis*, and *Cretogramma*) also have such fusion between forewing RP+MA and R, but with only one additional branch separated from R. In *Fiaponeura* and some kalligrammatid species (e.g. *Kallihemerobius*) there are two or more radial branches separated from R.

10. MA profusely branched from its base: (0) no; (1) yes.

11. MP occupying a large subtriangular area and pectinately branched from its base: (0) no; (1) yes.

12. Crossvenation: (0) modest; (1) extremely dense.

13. CuA with distal branches: (0) more than CuP branches; (1) fewer than CuP branches.

14. Outer gradate series of crossveins: (0) absent; (1) present.

15. Wing shape: (0) elongate and elliptical; (1) broad and subtriangular.

16. Forewing shape: (0) similar to hind wing; (1) different from hind wing.

17. Eyespot: (0) absent; (1) present.

18. Proximal nygma: (0) present; (1) absent. Comments: The presence of nygma, which is a thickened dot from wing membrane, could be plesiomorphic in Neuropterida. Thus, the absence of nygma either on proximal part or median part of wing (see below) is considered to be apomorphic.

19. Median nygma: (0) present; (1) absent.

20. Trichosors: (0) present; (1) absent.

Hind wing:

21. Sigmoid oblique MA stem: (0) present; (1) absent. Comments: A sigmoid oblique stem of hind wing MA is present in various groups of Neuropterida, probably being plesiomorphic. Thus, the absence of this veinlet is considered to be apomorphic and herein can be found in *Burmogramma*, *Burmopsychops*, and *Cretogramma*.

Head:

22. Three ocellus-like tubercles: (0) present; (1) absent.

23. Type of mouthparts: (0) chewing mandibulate; (1) long proboscid.

24. Mandible: (0) well developed; (1) strongly reduced.

25. Maxillary palp with terminal segment: (0) pointed distad; (1) inflated distad.
26. Sexual dimorphic antenna: (0) absent; (1) present.
27. Male antenna with flagellum: (0) filiform; (1) unipectinate; (2) bipectinate.
28. Female antenna with flagellum: (0) filiform; (1) moniliform.

Genitalia:
29. Male gonocoxites 9: (0) present as a pair of external sclerites; (1) present as one or a pair of internal sclerites.
30. Male gonocoxites 10: (0) paired; (1) unpaired, amalgamated with sternum 9; (2) absent.
31. Female tergum 9 strongly expanded posteriad, forming valvate structure together with ectoprocts: (0) absent; (1) present.
32. Female tergum 9 feebly sclerotized dorsally, with a pair of elongated lateral valves: (0) absent; (1) present.
33. Female gonocoxites 9: (0) not elongated; (1) elongated.

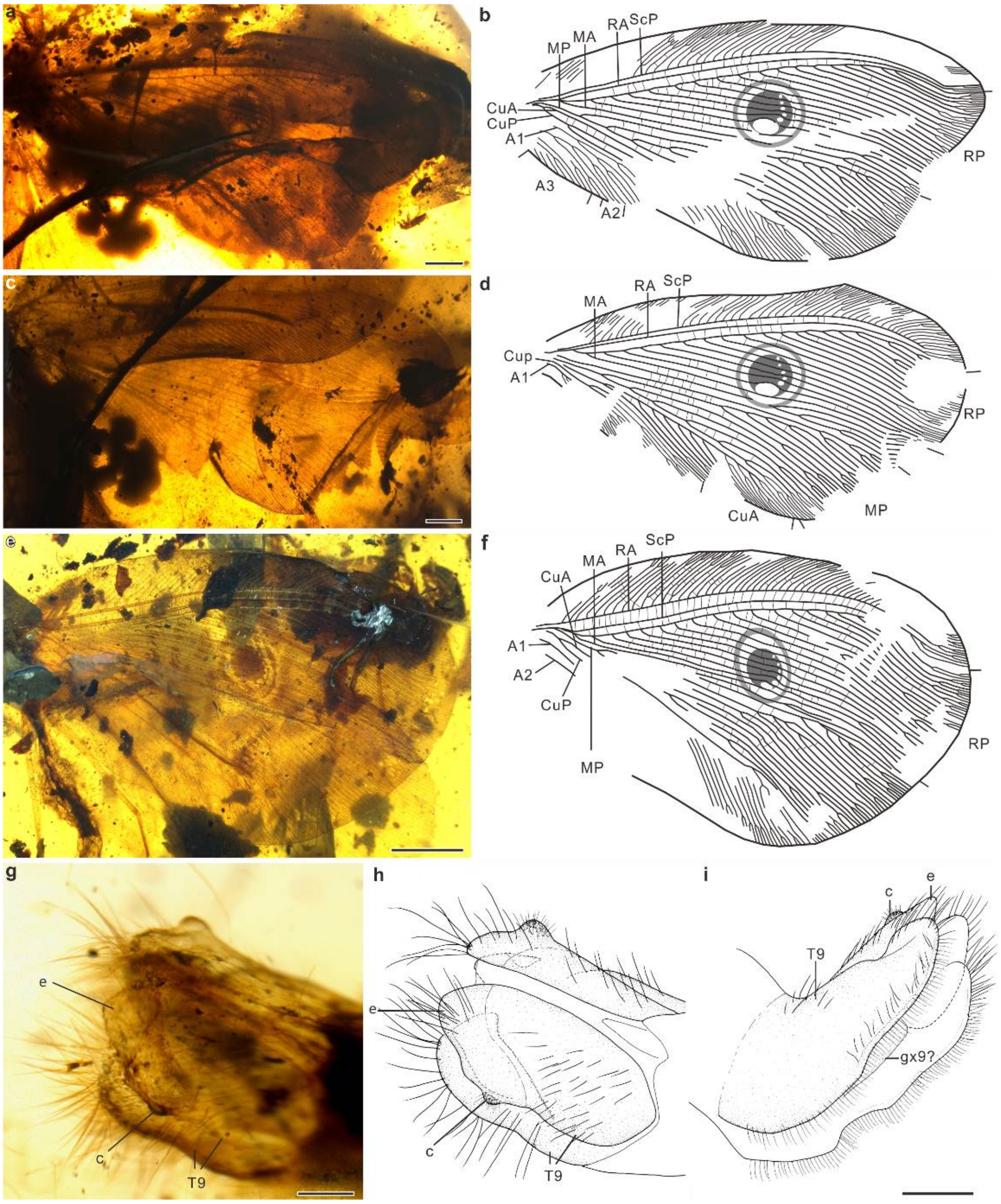

Supplementary Fig. 1 | *Burmogramma liui* from late Cretaceous Burmese amber. **a-d,** Holotype NIGP164471, male, forewing (**a, b**) and hindwing (**c, d**). **e-i,** Paratype NIGP164472, female, forewing (**e, f**) and genitalia (**g, h, i**). All wings aligned horizontally with apex to the right. **c**, callus cercus; e, ectoproct; gx, gonocoxite; T, tergum. Both (**a**) and (**b**) to scale. Both (**c**) and (**d**) to scale. Both (**e**) and (**f**) to scale. Both (**g**) and (**h**) to scale. Scale bars, 2 mm (**a–d**), 5 mm (**e–f**), 0.5 mm (**g, h**), and 1 mm (**i**).

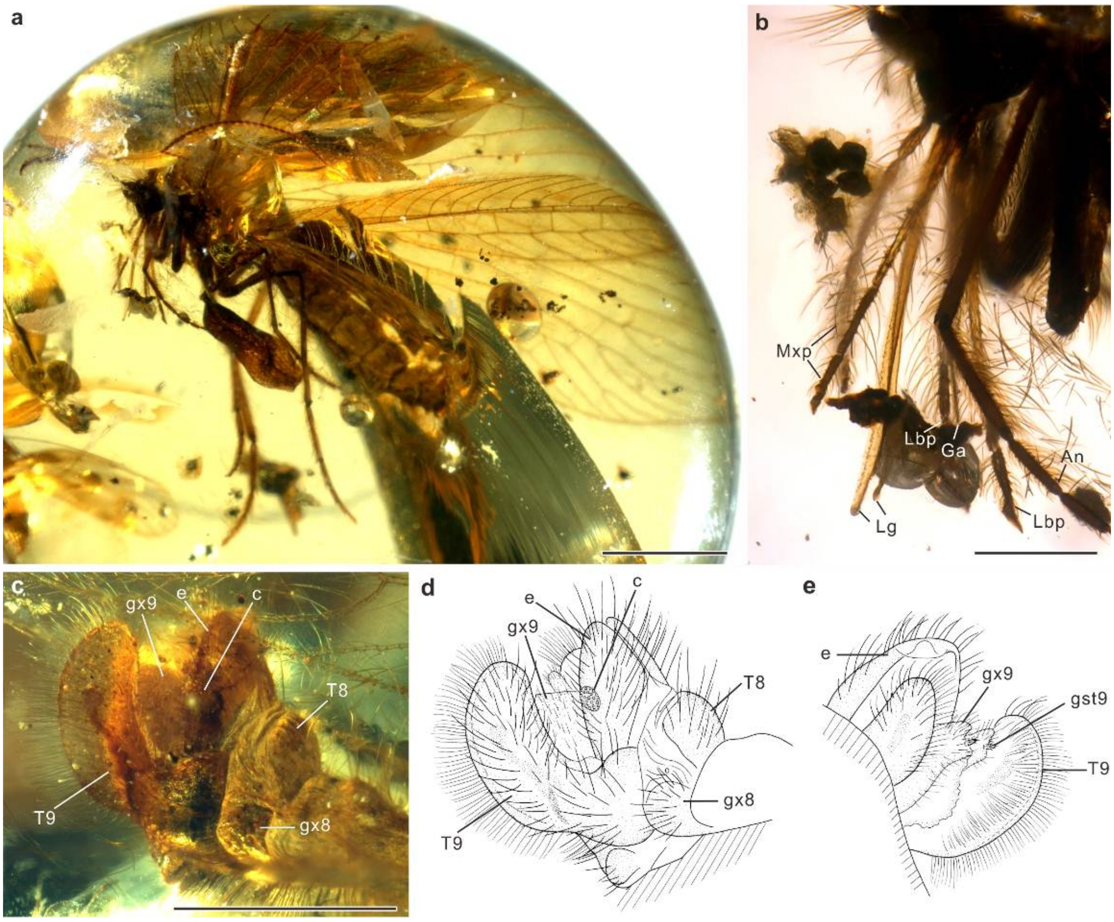

Supplementary Fig. 2 | *Burmopsychops limoae* from late Cretaceous Burmese amber. Specimen NIGP164485, female, habitus (**a**), mouthparts (**b**), genitals (**c–e**). An, antennae; c, callus cercus; e, ectoproct; Ga, galea; gst, gonostyl; gx, gonocoxite; Lbp, labial palp; Lg, ligula; Mxp, maxillary palp; T, tergum. (**c**), (**d**), and (**e**) to scale. Scale bars, 2 mm (**a**), 0.5 mm (**b**), 1 mm (**c–e**).

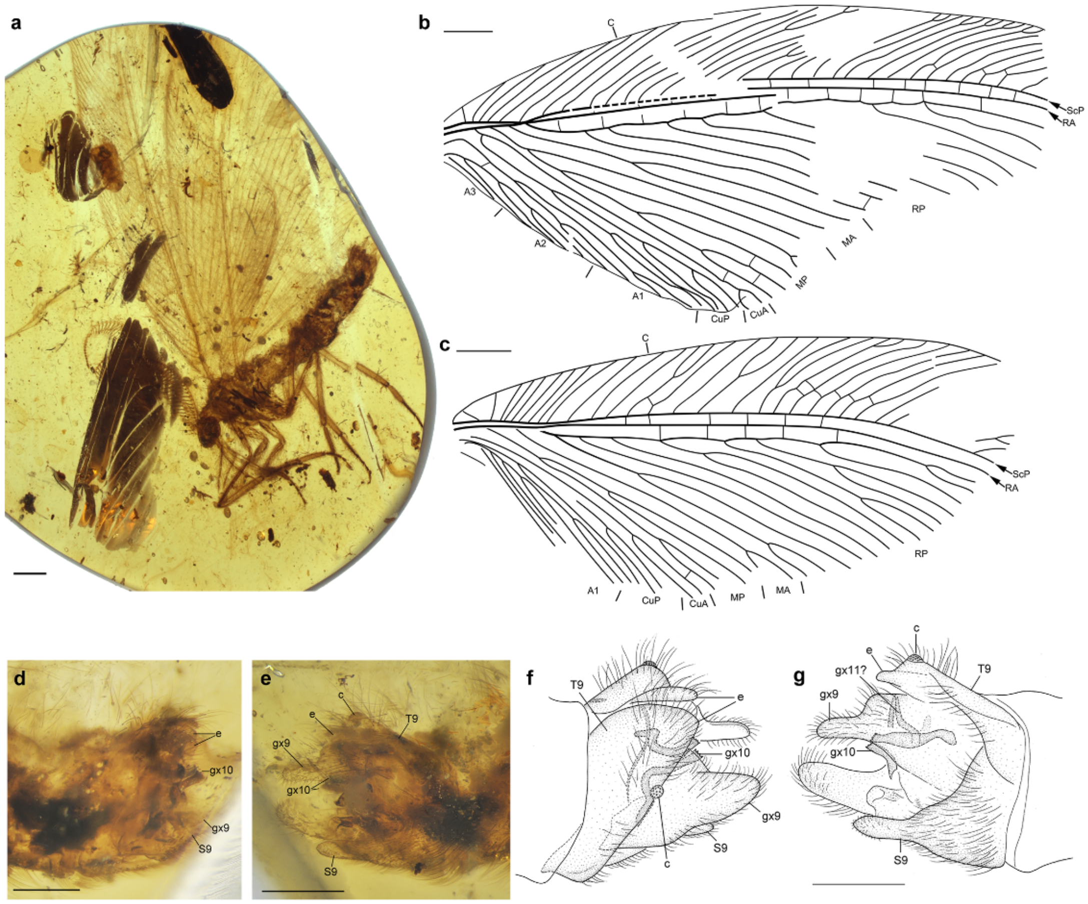

Supplementary Fig. 3 | *Burmopsychops labandeirai* from late Cretaceous Burmese amber. Holotype, NIGP164486, male, habitus (**a**), fore- and hind wing (**b, c**) and genitalia (**d–g**). c, callus cercus; e, ectoproct; gx, gonocoxite; S, sternum; T, tergum. Both (**f**) and (**g**) to scale. Scale bars, 1 mm (**a–c**), 0.5 mm (**d–g**).

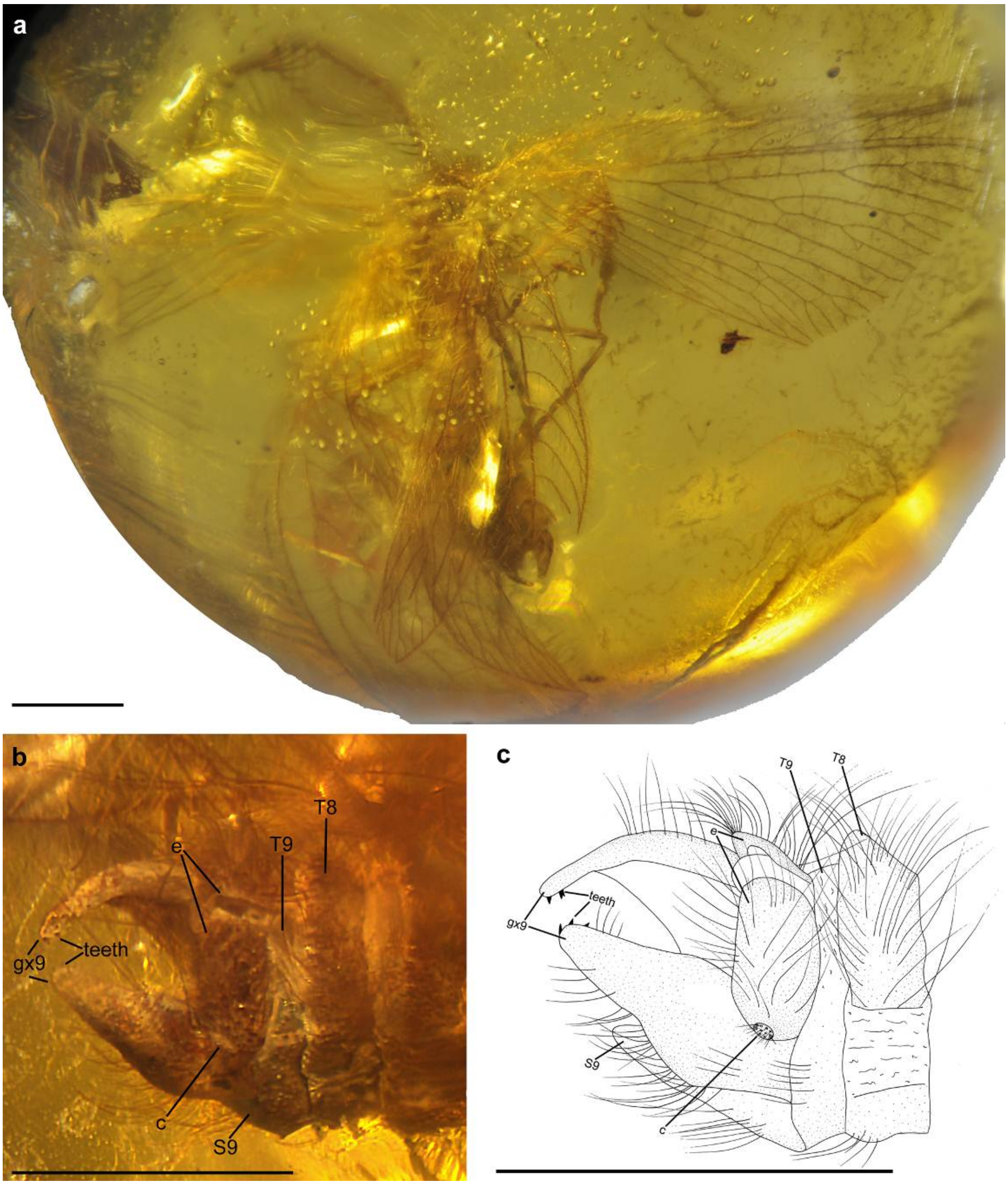

Supplementary Fig. 4 | *Cretanallachius magnificus* from late Cretaceous Burmese amber. Specimen EMTG BU-002169, male, habitus (**a**) and genitalia (**b, c**). c, callus cercus; e, ectoproct; gx, gonocoxite; S, sternum; T, tergum. Scale bars, 1 mm.

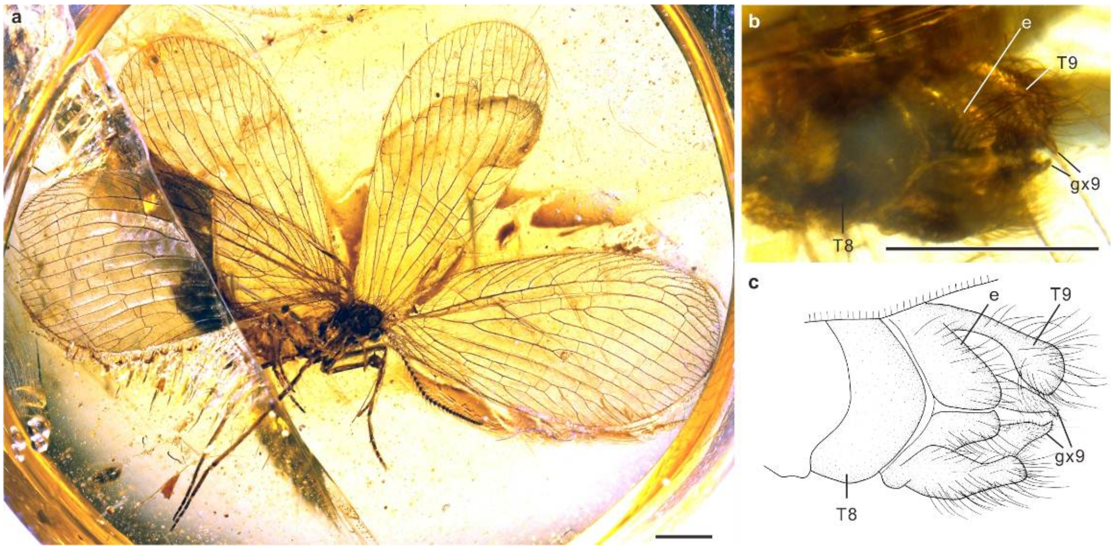

Supplementary Fig. 5 | *Cretanallachius magnificus* from late Cretaceous Burmese amber. Specimen NIGP164487, female, habitus (**a**) and genitalia (**b, c**). e, ectoproct; gx, gonocoxite; T, tergum. Both (**b**) and (**c**) to scale. Scale bars, 1 mm.

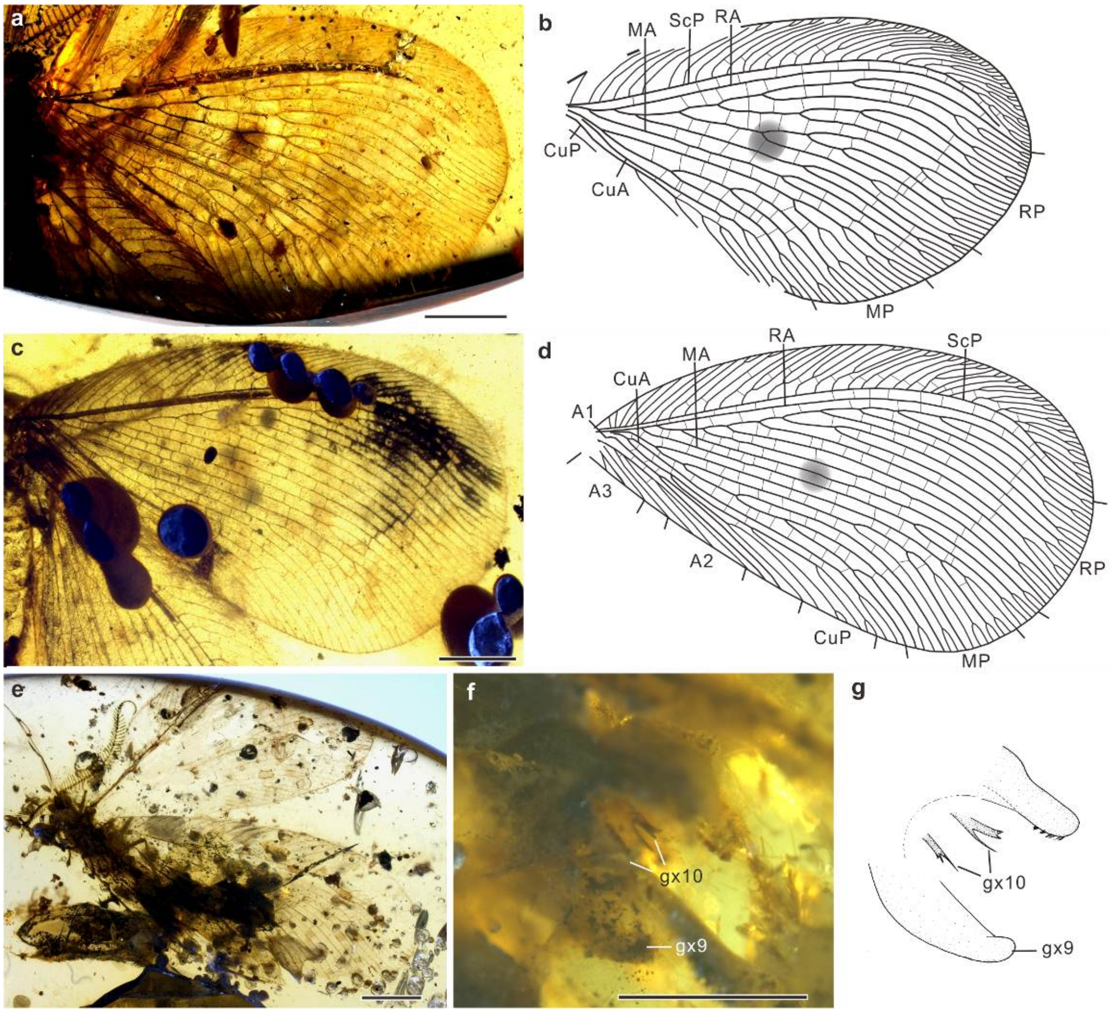

Supplementary Fig. 6 | *Cretogramma engeli* from late Cretaceous Burmese amber. **a, b,** Holotype, NIGP164481, male, forewing. **c, d,** Paratype, NIGP164483, female, forewing. **e-g,** NIGP164482, male, habitus (**e**) and genitalia (**f, g**). All wings aligned horizontally with apex to the right. gx, gonocoxite. Both (**a**) and (**b**) to scale. Both (**c**) and (**d**) to scale. Both (**f**) and (**g**) to scale. Scale bars, 2 mm (**a–e**), 0.5 mm (**f, g**).

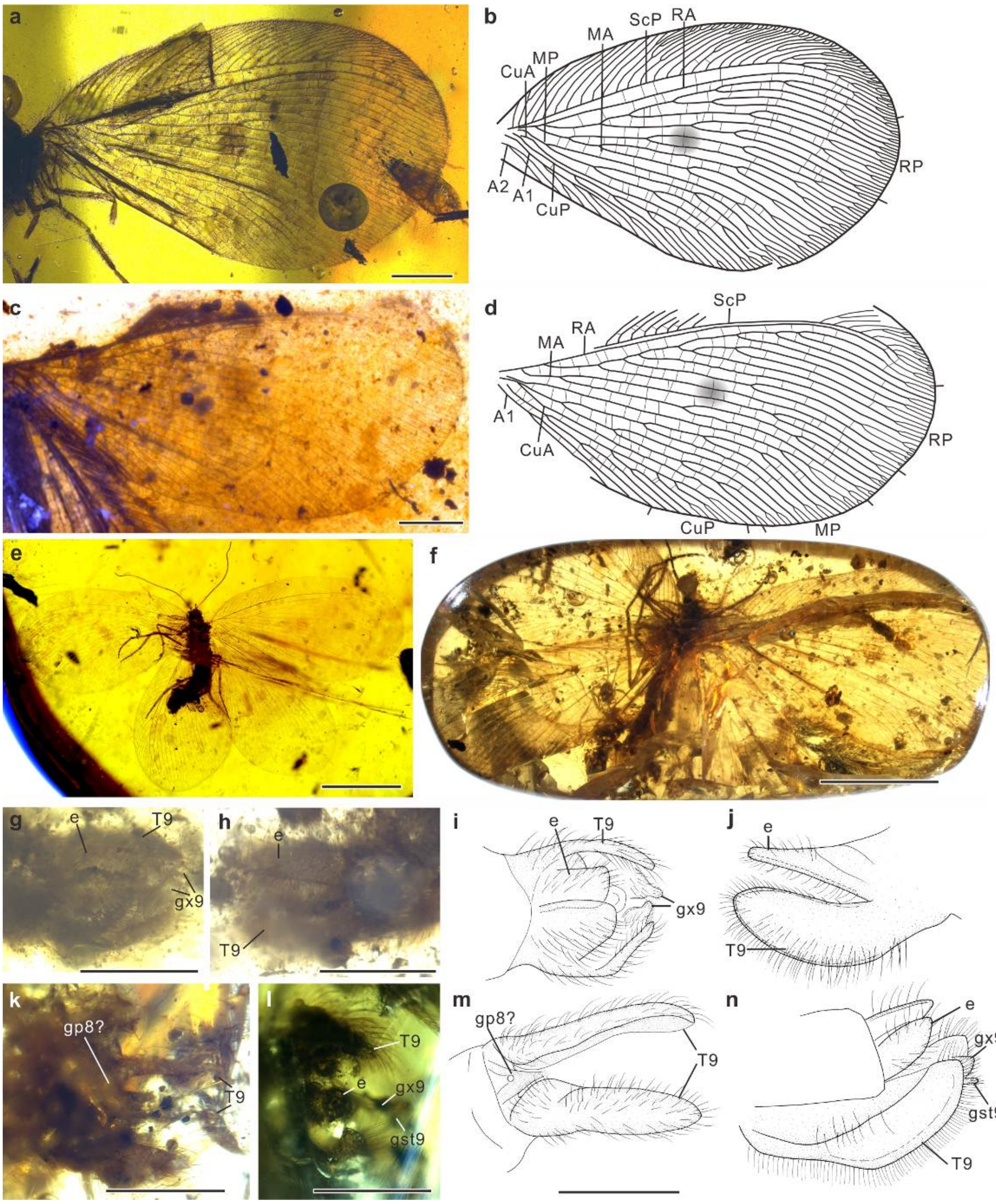

Supplementary Fig. 7 | *Oligopsychopsis groehni* from late Cretaceous Burmese amber. a, b, NIGP164476, male, forewing. **c, d,** NIGP164477, female, forewing. **e,** CAU-BA-XF-18002, female. **f, k–n,** NIGP164478, female, habitus (**f**) and genitalia (**k–n**). **g–j,** NIGP164479, female, genitals. All wings aligned horizontally with apex to the right. e, ectoproct; gp, gonapophysis; gst, gonostyli; gx, gonocoxite; T, tergum. Both (**a**) and (**b**) to scale. Both (**c**) and (**d**) to scale. (**i**), (**j**), (**m**), and (**n**) to scale. Scale bars, 2 mm (**a–d**), 5 mm (**e–f**), 1 mm (**g–n**).

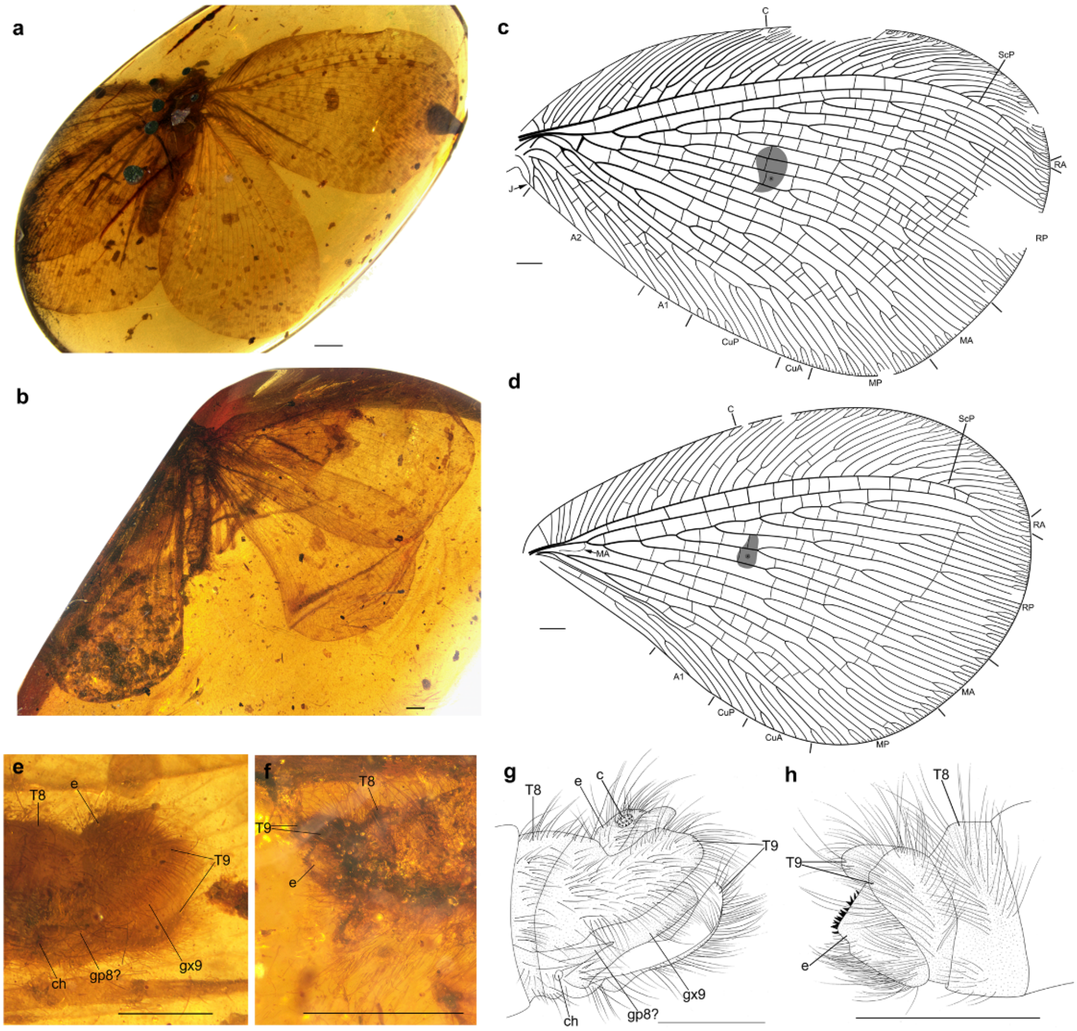

Supplementary Fig. 8 | *Oligopsychopsis grandis* from late Cretaceous Burmese amber. a, c, d, e, g, Holotype, CAM BA-0010, female, habitus (**a**), fore- and hind wing (**c, d**), and genitalia (**e, g**). **b, f, h,** Paratype, CAM BA-0011, male, habitus (**b**) and genitalia (**f, h**). c, callus cercus; ch, copulatory hole; e, ectoproct; gp, gonapophysis; gx, gonocoxite; T, tergum. Scale bars, 2 mm (**a**), 1 mm (**b–h**).

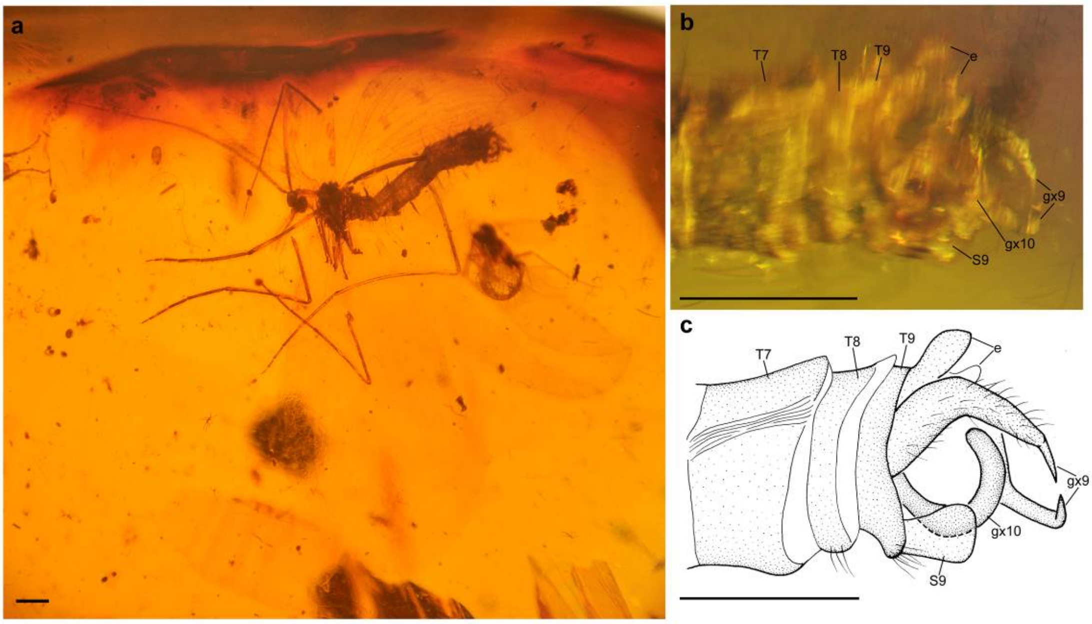

Supplementary Fig. 9 | *Fiaponeura penghiani* from late Cretaceous Burmese amber. Specimen NIGP168202, male, habitus (**a**), genitalia (**b–c**). e, ectoproct; gx, gonocoxite; S, sternum; T, tergum. Scale bars, 1 mm.

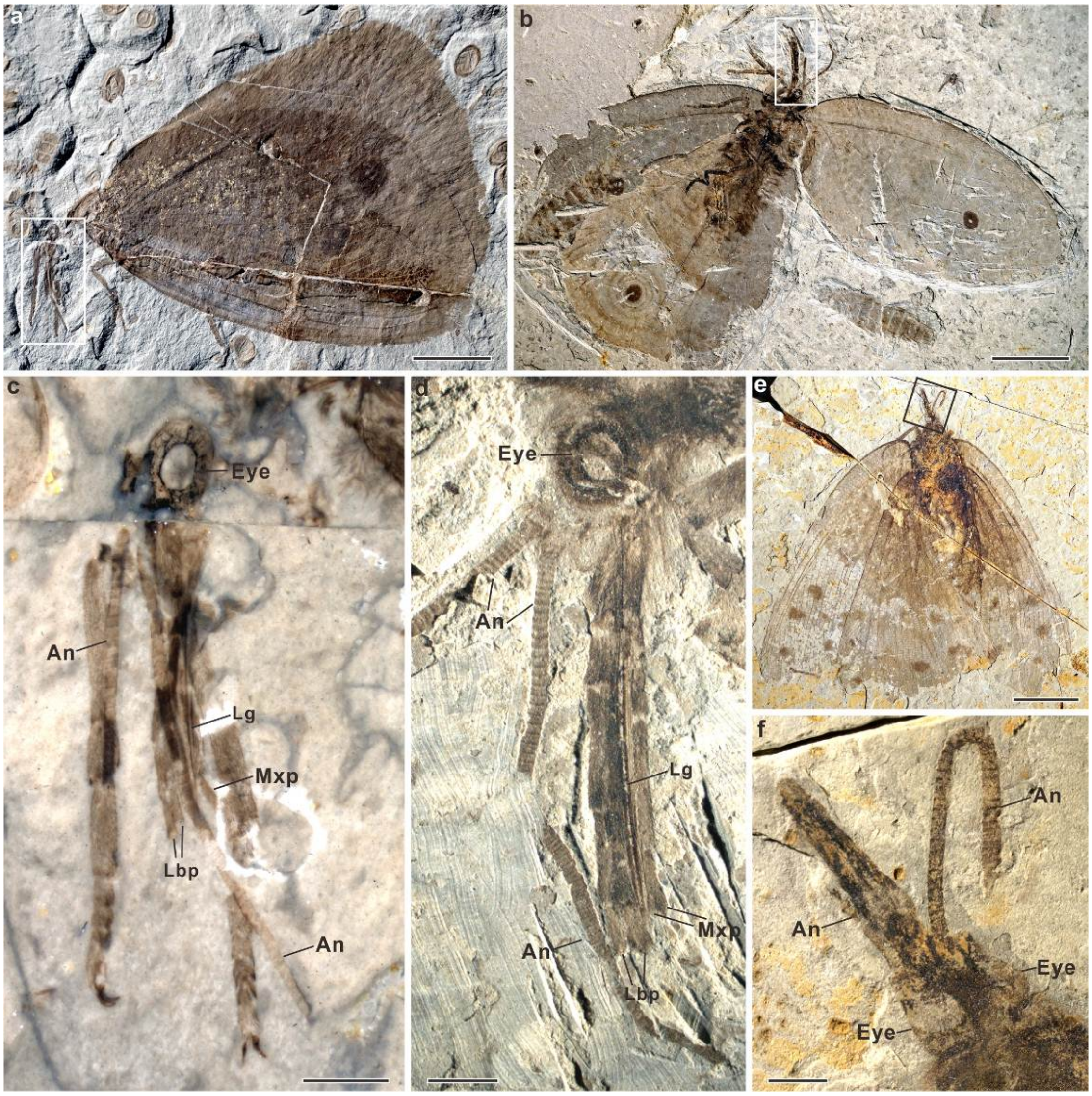

Supplementary Fig. 10 | Middle Jurassic and early Cretaceous kalligrammatids from northeastern China. a, **c,** *Kalligramma* sp. from the Middle Jurassic of Daohugou, NIGP164470. **c**, Enlargement of mouthparts in (**a**). **b**, **d,** *Oregramma* sp. from the Lower Cretaceous of Liutiaogou, STMN45-1614. **d**, Enlargement of mouthparts in (**b**). **e, f,** *Oregramma* sp. from the Lower Cretaceous of Liutiaogou, STMN45-1624. **f,** Enlargement of mouthparts in (**e)**. An, antennae; Eye, compound eye; Lg: ligula; Lbp: labial palp; Mxp: maxillary palp. Scale bars, 10 mm (**a**), 20 mm (**b, e**), 2 mm (**c, d, f**).

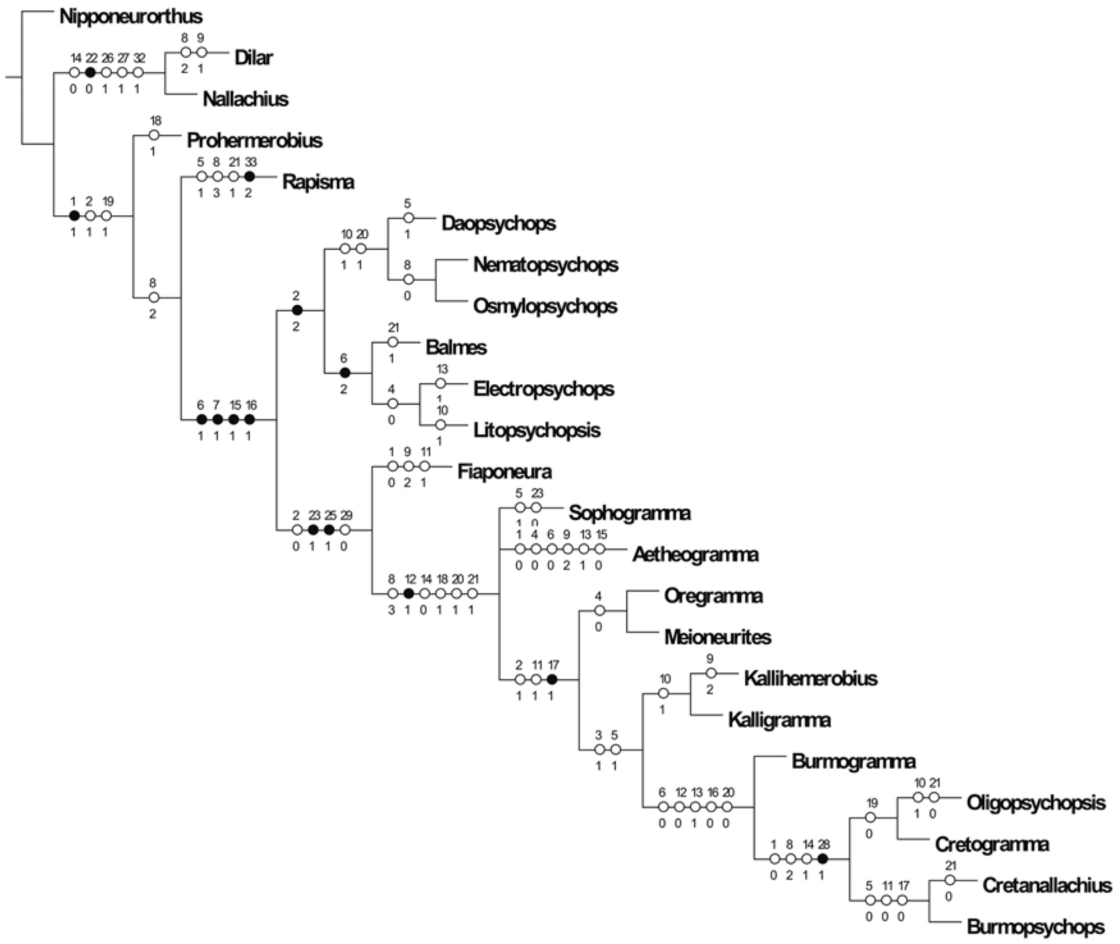

Supplementary Fig. 11 | Preferred most parsimonious tree generated from matrix 1.

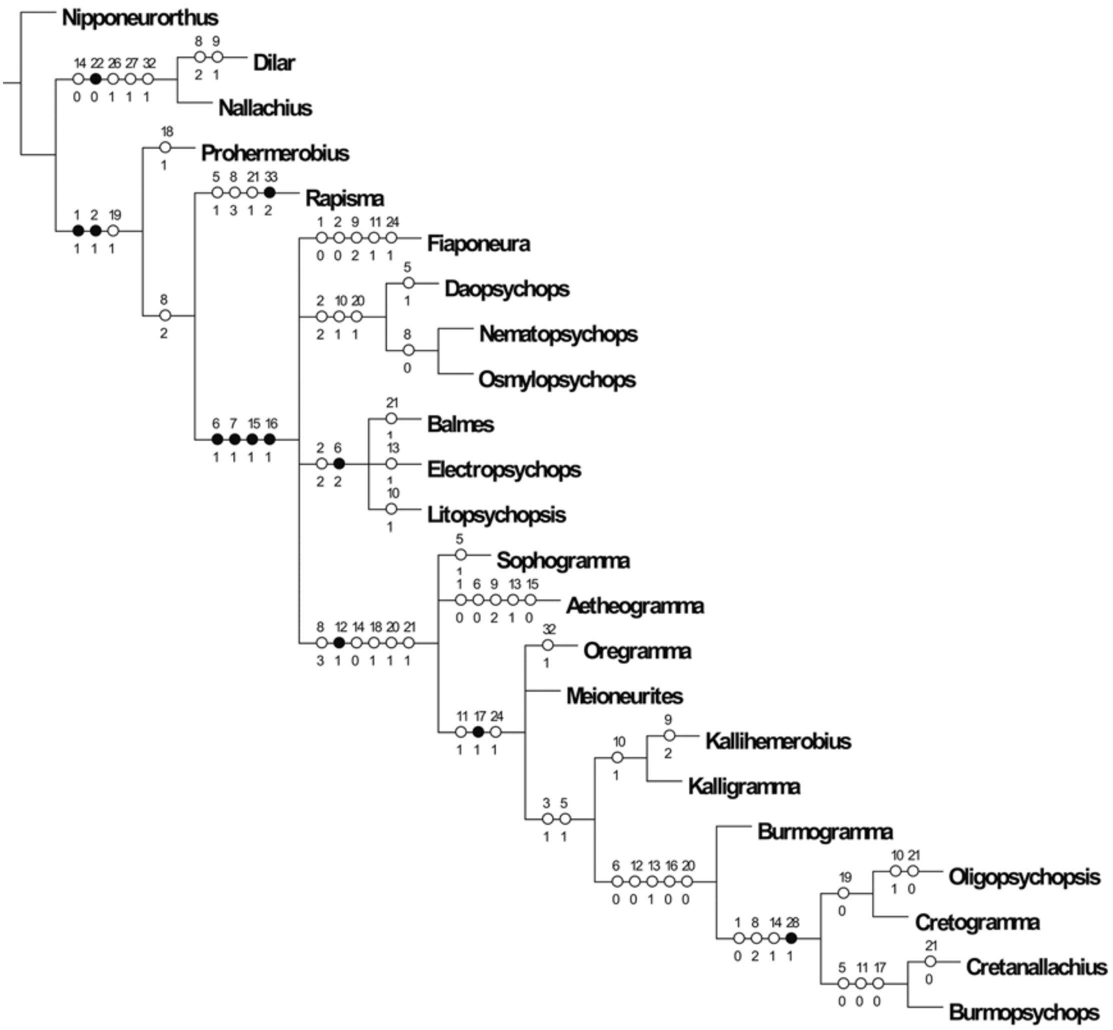

Supplementary Fig. 12 | Strict consensus tree of eight most parsimonious trees generated from matrix 1.

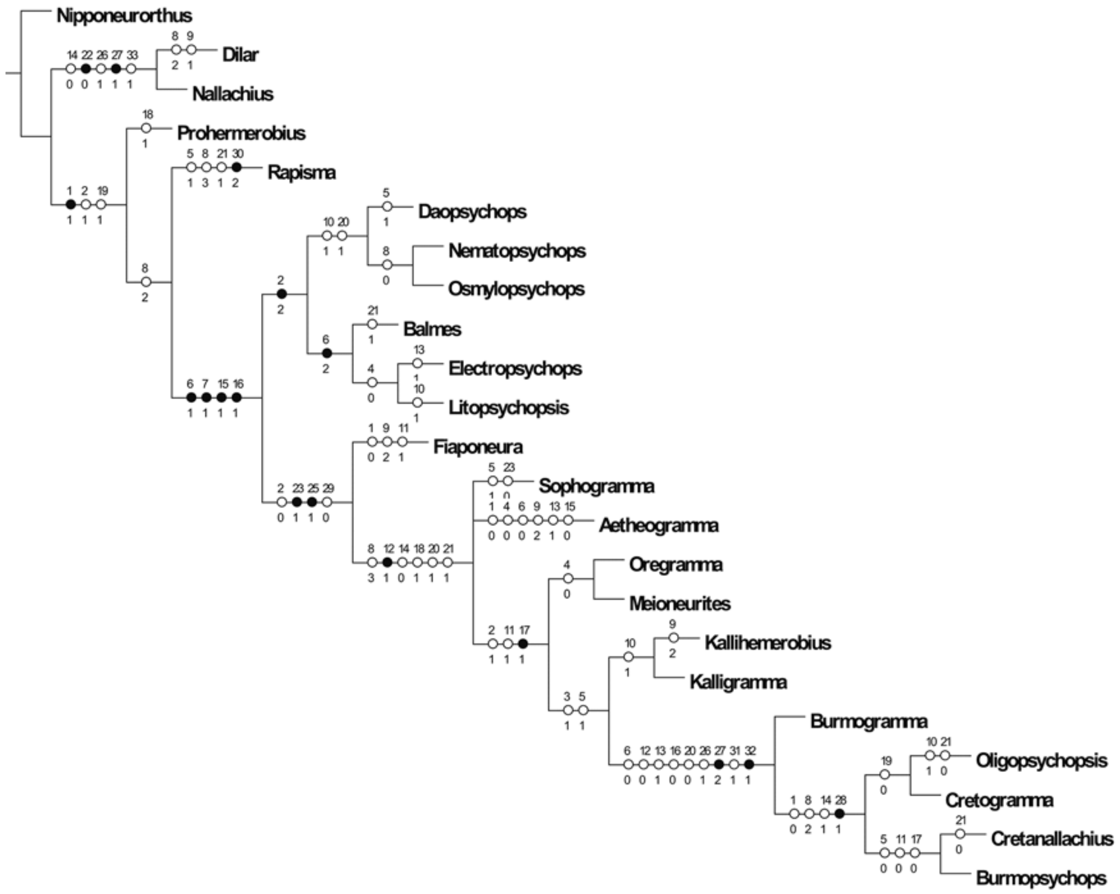

Supplementary Fig. 13 | Preferred most parsimonious tree generated from matrix 2.

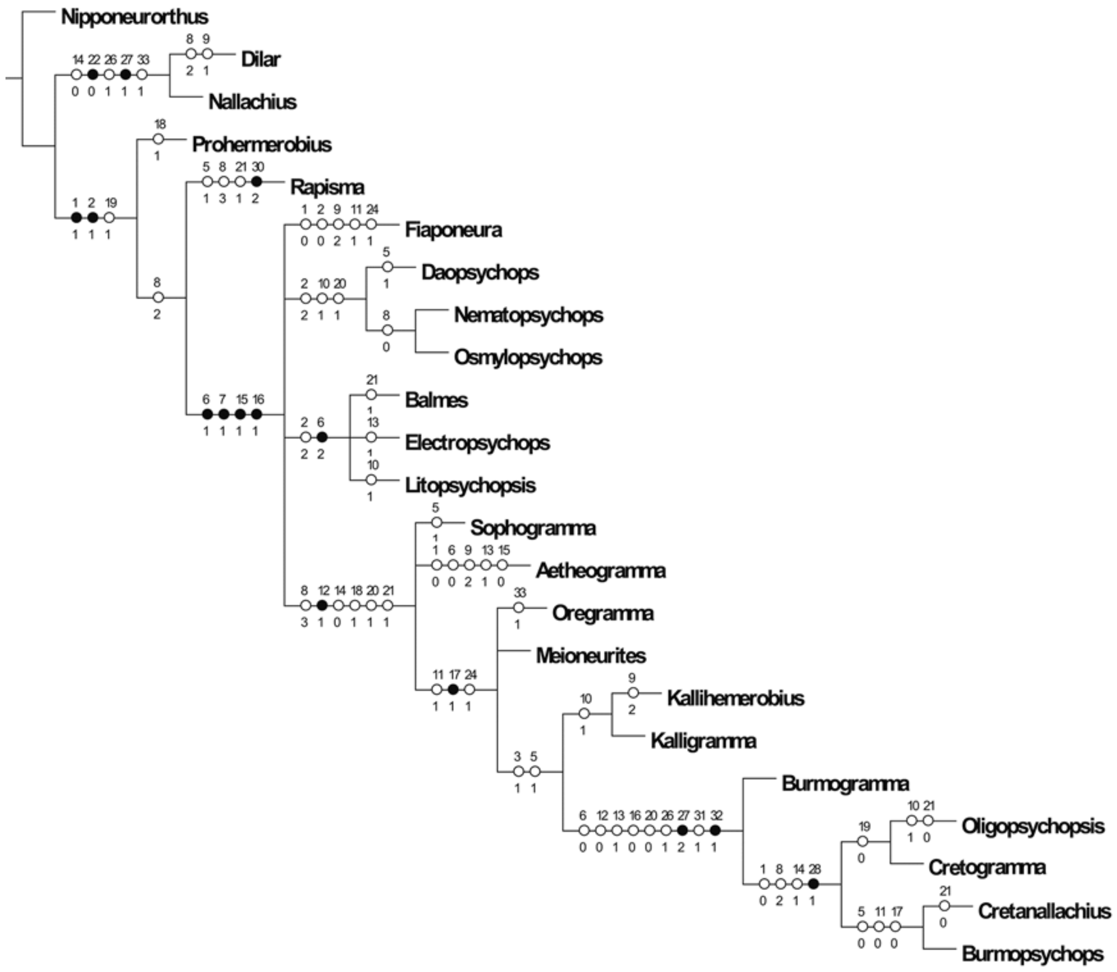

Supplementary Fig. 14 | Strict consensus tree of eight most parsimonious trees generated from matrix 2.

Supplementary Table 1 | Measurements of 27 kalligrammatid lacewings. FL, forewing length; MPL, maxillary palpus length; PL, proboscid length; LPL, labial palpus length; n, absent or unknown.

Number	Taxa	FL(mm)	MPL(mm)	PL(mm)	LPL(mm)	Gender	Note
NIGP164470	*Kalligramma* sp.	50.8	6.4	6.3	6.4	unknown	Daohugou; Middle Jurassic
STMN45-1624	*Oregramma* sp.	~83	7.5	7.2	7.5	unknown	Liutiaogou; Lower Cretaceous
STMN45-1614	*Oregramma* sp.	74.8	12.6	12.1	12.6	unknown	Liutiaogou; Lower Cretaceous
NIGP164471	*Burmogramma liui* sp. nov.	26.0	3.4	3.2	3.6	male	Mouthparts disarticulated
NIGP164472	*Burmogramma liui* sp. nov.	31.8	n	n	n	female	Mouthparts invisible
NIGP164473	*Burmogramma liui* sp. nov.	~30	3.4	3.2	3.8	female	
NIGP164474	*Burmogramma liui* sp. nov.	~30	n	n	n	female	Mouthparts lost
NIGP164475	*Burmogramma liui* sp. nov.	~32	3.4	3.2	3.7	female	
NIGP164485	*Burmopsychops limoae*	~10	1.4	1.1	1.6	female	
NIGP164486	*Burmopsychops labandeirai* sp. nov.	~13	1.8	1.7	2.1	male	
EMTG BU-002169	*Cretanallachius magnificus*	~7	n	n	n	male	Mouthparts poorly visible
EMTG BU-002266	*Cretanallachius magnificus*	~7	n	n	n	male	Mouthparts poorly visible
NIGP164487	*Cretanallachius magnificus*	7.3	0.7	0.6	0.8	female	
CAU-BA-XF-18003	*Cretanallachius magnificus*	7.6	0.7	0.6	0.8	female	

NIGP164481	*Cretogramma engeli* sp. nov.	11.6	1.4	1.3	1.6	male	
NIGP164482	*Cretogramma engeli* sp. nov.	12.0	n	n	n	male	Mouthparts poorly visible
NIGP164483	*Cretogramma engeli* sp. nov.	13.5	1.4	1.3	1.7	female	
NIGP164484	*Cretogramma engeli* sp. nov..	13.8	1.4	1.3	1.7	female	
NIGP164476	*Oligopsychopsis groehni*	n	n	n	n	male	Mouthparts and forewings lost
NIGP164477	*Oligopsychopsis groehni*	14.4	1.5	1.4	1.8	female	
NIGP164478	*Oligopsychopsis groehni*	~15	n	n	n	female	Mouthparts poorly visible
NIGP164479	*Oligopsychopsis groehni*	14.6	1.5	1.4	1.7	female	
CAU-BA-XF-18002	*Oligopsychopsis groehni*	14.8	1.7	1.5	2.0	female	
CAU-BA-WN-18001	*Oligopsychopsis groehni*	~15	1.6	1.5	1.9	female	
CAM BA-0010	*Oligopsychopsis grandis* sp. nov.	19.0	2.0	1.9	2.3	female	
CAM BA-0011	*Oligopsychopsis grandis* sp. nov.	14.0	n	n	n	male	Mouthparts poorly visible
NIGP164480	*Oligopsychopsis grandis* sp. nov.	20.9	n	n	n	female	Head lost

Supplementary Table 2 | Proboscid length of Mesozoic long-proboscid insects. PL, proboscid length.

Taxa	PL (mm)	Age	Reference
**Neuroptera: Kalligrammatidae**			
Affinigramma myrioneura	13.9	Middle Jurassic	Labandeira et al., 2016[8]
Kallihemerobius aciedentatus	9.1-9.8	Middle Jurassic	Labandeira et al., 2016[8]
Kallihemerobiinae gen. indet.	10.3	Middle Jurassic	Labandeira et al., 2016[8]
Kalligramma brachyrhyncha	11.2	Middle Jurassic	Labandeira et al., 2016[8]
Kalligramma circularia	5.4-8.5	Middle Jurassic	Labandeira et al., 2016[8]
Kalligramma sp.	>10	Middle Jurassic	Labandeira et al., 2016[8]
Kalligramma sp.	6.3	Middle Jurassic	This study
Meioneurites spectabilis	~8	Late Jurassic	Engel, 2005[9]
Abrigramma calophleba	10.7	Early Cretaceous	Labandeira et al., 2016[8]
Ithigramma multinervia	9.8	Early Cretaceous	Labandeira et al., 2016[8]
Ithigramma sp.	>9.4	Early Cretaceous	Labandeira et al., 2016[8]
Oregramma aureolusa	>18	Early Cretaceous	Labandeira et al., 2016[8]
Oregramma illecebrosa	16.1	Early Cretaceous	Labandeira et al., 2016[8]
Oregramma sp.	7.6	Early Cretaceous	Labandeira et al., 2016[8]
Oregramma sp.	7.2	Early Cretaceous	This study
Oregramma sp.	12.1	Early Cretaceous	This study
Burmogramma liui	3.2	Late Cretaceous	This study
Burmopsychops limoae	1.1	Late Cretaceous	This study
Burmopsychops labandeirai	1.7	Late Cretaceous	This study
Cretanallachius magnificus	0.6	Late Cretaceous	This study
Cretogramma engeli	1.3	Late Cretaceous	This study
Oligopsychopsis penniformis	1.6	Late Cretaceous	Chang et al., 2018[7]
Oligopsychopsis groehni	1.4-1.5	Late Cretaceous	This study
Oligopsychopsis grandis	1.9	Late Cretaceous	This study
**Family Incertae sedis**			
Fiaponeura penghiani	0.5	Late Cretaceous	Lu et al., 2016[2]
**Neuroptera: Sisyridae**			
Paradoxosisyra groehni	0.7-1.0	Late Cretaceous	This study
**Mecoptera: Mesopsychidae**			
Lichnomesopsyche gloriae	8.9-10.1	Middle Jurassic	Ren et al., 2009[10]
Lichnomesopsyche daohugouensis	8.8	Middle Jurassic	Ren et al., 2009[10]
Vitimopsyche kozlovi	9.0	Early Cretaceous	Ren et al., 2009[10]
**Mecoptera: Pseudopolycentropodidae**			

Pseudopolycentropus daohugouensis	1.6	Middle Jurassic	Grimaldi et al., 2005[11]
Pseudopolycentropus novokshonovi	1.5	Middle Jurassic	Ren et al., 2009[10]
Pseudopolycentropus janeannae	1.6	Middle Jurassic	Ren et al., 2009[10]
Pseudopolycentropus latipennis	>1.8	Late Jurassic	Ren et al., 2009[10]
Parapolycentropus burmiticus	1.3	Late Cretaceous	Grimaldi & Johnston, 2014[12]
Parapolycentropus paraburmiticus	0.6-1.0	Late Cretaceous	Grimaldi & Johnston, 2014[12]
**Mecoptera: Aneuretopsychidae**			
Aneuretopsyche minima	4.7	Late Jurassic	Rasnitsyn & Kozlov, 1990[13]
Aneuretopsyche rostrata	7.3	Late Jurassic	Rasnitsyn & Kozlov, 1990[13]
Jeholopsyche liaoningensis	5.8-6.8	Early Cretaceous	Ren et al., 2009[10]
Jeholopsyche completa	3.5	Early Cretaceous	Qiao et al., 2012[14]
Jeholopsyche bella	~4	Early Cretaceous	Qiao et al., 2012[14]
Jeholopsyche maximus	14.5	Early Cretaceous	Qiao et al., 2012[14]
**Diptera: Brachycera: Athericidae**			
Palaepangonius eupterus	>2	Early Cretaceous	Ren, 1998[15]; Zhang, 2012[16]
**Diptera: Brachycera: Tabanidae**			
Eopangonius pletus	~3	Early Cretaceous	Ren, 1998[15]
**Diptera: Brachycera: Rhagionidae**			
Orsobrachyceron chinensis	1.2	Early Cretaceous	Ren, 1998[15]
Oiobrachyceron limnogenus	1.0	Early Cretaceous	Ren, 1998[15]
**Diptera: Brachycera: Nemestrinidae**			
Protonemestrius jurasicus	>5	Early Cretaceous	Ren, 1998[15]
Florinemestrius pulcherrimus	>3.1	Early Cretaceous	Ren, 1998[15]
**Diptera: Brachycera: Zhangsolvidae**			
Zhangsolva cupressa	~6	Early Cretaceous	Zhang et al., 1993[17]
Cratomyia macrorrhyncha	>4.5	Early Cretaceous	Mazzarolo & Amorim, 2000[18]
Cratomyia cretacica	~1.3	Early Cretaceous	Wilkommen & Grimaldi, 2007[19]
Buccinatormyia magnifica	3.85	Early Cretaceous	Arillo et al., 2015[20]
Buccinatormyia soplaensis	>1.07	Early Cretaceous	Arillo et al., 2015[20]
Linguatormyia teletacta	4.37	Late Cretaceous	Arillo et al., 2015[20]

Supplementary References

1. Makarkin, V. N. New taxa of unusual Dilaridae (Neuroptera) with siphonate mouthparts from the mid-Cretaceous Burmese amber. *Cretac. Res.* **74**, 11–22 (2017).
2. Lu, X., Zhang, W. & Liu, X. New long-proboscid lacewings of the mid-Cretaceous provide insights into ancient plant-pollinator interactions. *Sci. Rep.* **6**, 25382 (2016).
3. Huang, D., Azar, D., Cai, C., Garrouste, R. & Nel, A. The first Mesozoic pleasing lacewing (Neuroptera: Dilaridae). *Cretac. Res.* **56**, 274–277 (2015).
4. Gao, T. et al. Convergent evolution of ramified antennae in insect lineages from the Early Cretaceous of Northeastern China. *Proc. R. Soc. B* **283**, 20161448 (2016).
5. Oswald, J. D. Revision and cladistic analysis of the world genera of the Family Hemerobiidae (Insecta: Neuroptera). *J. New York Entomol. Soc.* **101**, 143–299 (1993).
6. Bakkes, D. K., Mansell, M. W. & Sole C. L. Phylogeny and historical biogeography of silky lacewings (Neuroptera: Psychopsidae). *Syst. Entomol.* **43**, 43–55 (2018).
7. Chang, Y., Fang, H., Shih, C., Ren, D. & Wang, Y. Reevaluation of the subfamily Cretanallachiinae Makarkin, 2017 (Insecta, Neuroptera) from Upper Cretaceous Myanmar amber. *Cretac. Res.* **84**, 533–539 (2018).
8. Labandeira, C. C. et al. The evolutionary convergence of mid-Mesozoic lacewings and Cenozoic butterflies. *Proc. R. Soc. B* **283**, 20152893 (2016).
9. Engel, M. S. A remarkable kalligrammatid lacewing from the upper Jurassic of Kazakhstan (Neuroptera: Kalligrammatidae). *Trans. Kans. Acad. Sci.* **108**, 59–62 (2005).
10. Ren, D. et al. A probable pollination mode before angiosperms: Eurasian, long-proboscid scorpionflies. *Science* **326**, 840–847 (2009).
11. Grimaldi, D., Zhang, J., Fraser, N. C. & Rasnitsyn, A. Revision of the bizarre Mesozoic scorpionflies in the Pseudopolycentropodidae (Mecopteroidea). *Insect Syst. Evol.* **36**, 443–458 (2005).
12. Grimaldi, D. & Johnston, M. A. The long-tongued Cretaceous scorpionfly Parapolycentropus Grimaldi and Rasnitsyn (Mecoptera: Pseudopolycentropodidae): New data and interpretations. *Am. Mus. Novit.* **3793**,

1–24 (2014).

13. Rasnitsyn, A. P. & Kozlov, M. V. A new group of fossil insects: scorpionflies with cicada and butterfly adaptations. *Dokl. Akad. Nauk SSS*R **310**, 973–976 (1990).
14. Qiao, X., Shih, C. K. & Ren, D. Three new species of aneuretopsychids (Insecta: Mecoptera) from the Jehol Biota, China. *Cretac. Res.* **36**, 146–150 (2012).
15. Ren, D. Late Jurassic Brachycera from Northeastern China (Insecta: Diptera). *Acta Zootaxon. Sin.* **23**, 65–83 (1998).
16. Zhang, J. New horseflies and water snipe-flies (Diptera: Tabanidae and Athericidae) from the Lower Cretaceous of China. *Creatac. Res.* **36**, 1–5 (2012).
17. Zhang, J., Zhang, S. & Li, L. Y. Mesozoic gadflies (Insecta: Diptera). *Acta Palaeontol. Sin.* **32**, 662–672 (1993).
18. Mazzarolo, L. A. & Amorim, D. S. Cratomyia macrorrhyncha, a Lower Cretaceous brachyceran fossil from the Santana Formation, Brazil, representing a new species, genus and family of the Stratiomyomorpha (Diptera). *Insect Syst. Evol.* **31**, 91–102 (2000).
19. Willkommen, J. & Grimaldi, D. A. Diptera: true flies, gnats and crane flies. (The Crato Fossil Beds of Brazil) 369–387 (2007).
20. Arillo, A. et al. Long-proboscid brachyceran flies in Cretaceous amber (Diptera: Stratiomyomorpha: Zhangsolvidae). *Syst. Entomol.* **40**, 242–267 (2015).
