# Supplementary material for: High niche diversity in Mesozoic pollinating lacewings
Source: Nat Commun. 2018 Sep 17;9:3793. doi: 10.1038/s41467-018-06120-5 (PMC6141599; doi:10.1038/s41467-018-06120-5)
Supplement: Supplementary file 3 — Description of Additional Supplementary Files [file 41467_2018_6120_MOESM3_ESM.pdf]

Description of Additional Supplementary Files

File Name: Supplementary Data 1
Description: Matrix.

File Name: Supplementary Data 2
Description: Matrix with estimated codes.
